# Traumatic memories are directly associated with depression and generalized anxiety symptoms, independent of posttraumatic stress symptoms

**DOI:** 10.1016/j.ijchp.2026.100686

**Published:** 2026-04-22

**Authors:** Marcus Broughill, Sean Commins, Philip Hyland

**Affiliations:** Department of Psychology, Maynooth University, Kildare, Ireland

**Keywords:** Trauma, Memory, Posttraumatic stress disorder (PTSD), Depression, Generalized anxiety disorder (GAD)

## Abstract

**Background:**

Disturbances in memory following trauma exposure are central to the development of posttraumatic stress disorders. Whether traumatic memories directly or indirectly affect symptoms of other disorders like depression and generalized anxiety disorder (GAD) remains unclear. This study tested the hypothesis that traumatic memories are directly related to depression and GAD symptoms, independent of posttraumatic stress symptoms.

**Method:**

Cross-sectional data from quasi-representative samples from Ukraine (*N* = 2050) and the United Kingdom (*N* = 975) were analysed. Posttraumatic distress was measured according to the *ICD-11* description (i.e., posttraumatic stress disorder [PTSD] and disturbances in self-organization [DSO] symptoms).

**Results:**

Traumatic memories were strongly, positively, and significantly correlated with PTSD, DSO, depression, and GAD symptoms. Controlling for PTSD symptoms, traumatic memories remained positively (*p* < 0.001) associated with depression (*β* = 0.37– 0.41) and GAD (*β* = 0.35– 0.36) symptoms. Controlling for PTSD and DSO symptoms, associations remained positive and significant (*β* = 0.11–.21, *p* < 0.001).

**Conclusion:**

Traumatic memories were associated with depression and GAD symptoms, independent of the effects of posttraumatic stress symptoms. These findings are consistent with the proposition that traumatic memories are directly related to depression and GAD symptoms, as well as posttraumatic stress symptoms. Theoretical and clinical implications are discussed.

Trauma is defined as any extremely threatening or horrifying event typically involving a direct or indirect threat to life or physical safety (American Psychiatric Association, 2022; World Health Organization, 2019). Approximately 70% of people globally will experience a traumatic event (Benjet et al., 2016), and although trauma exposure increases risk for a range of psychological disorders (Hogg et al., 2023), it is central to the development of posttraumatic stress disorders (PTSDs). In the *Diagnostic and Statistical Manual of Mental Disorders* (*DSM-5-TR*: (American Psychiatric Association, 2022), PTSD is defined by four symptom clusters: intrusions, avoidance, negative alterations in cognition and mood, and alterations in arousal and reactivity. In contrast, the *International Classification of Diseases* (*ICD-11*: (World Health Organization, 2019) distinguishes PTSD and Complex PTSD (CPTSD) as separate disorders, with PTSD defined by three symptom clusters (re-experiencing in the here and now, avoidance, and sense of current threat) and CPTSD by six (three shared with PTSD plus affective dysregulation, negative self-concept, and disturbed relationships that are collectively termed ‘disturbances in self-organisation’ [DSO]).

Epidemiological surveys indicate that 7–8% of the general adult population meet diagnostic criteria for PTSD or CPTSD, regardless of the diagnostic system used (Cloitre et al., 2019; de Vries & Olff, 2009; Kessler et al., 1995; Kilpatrick et al., 2013). Thus, most people recover naturally after trauma with no long-term effects (Bonanno, 2004; Bonanno, 2005; Bonanno & Mancini, 2008). Consequently, PTSD has been described as a disorder where natural recovery fails to occur (Brewin et al., 2025; Yehuda & LeDoux, 2007). The mechanisms underlying this failure to recover are well understood (Brewin et al., 2025; Brewin & Ehlers, 2023; Yehuda et al., 2015), with a central role attributed to disturbances in episodic and autobiographical memory; forming the basis of the dual representation theory of PTSD (Brewin, 2014; Brewin & Burgess, 2014; Brewin et al., 1996; Brewin et al., 2010).

The dual representation theory proposes that PTSD develops due to a disconnect between contextual and sensory-based representations of a traumatic event within memory. During a traumatic event, an individual experiences extreme threat or horror, feelings that are accompanied by the release of catecholamines (e.g., epinephrine, norepinephrine) and stress-related hormones (e.g., cortisol) that can inhibit hippocampal activity (responsible for processing contextual information such as time, space, and sequence of the event) and increase activity in the amygdala (involved in processing sensory and emotional information). Under normal conditions, contextual and sensory-emotional information is integrated, forming coherent episodic memories within autobiographical memory that can be voluntarily recalled and experienced as past events. However, under extreme stress this integration is disrupted, resulting in fragmented and incoherent episodic memories that are difficult to recall voluntarily, and are usually triggered involuntarily by cues resembling aspects of the traumatic event (Brewin & Field, 2024). When cued, these memories typically involve vivid sensory and emotional experiences with limited or no contextual information resulting in a felt sense that the event is being relived in the present moment.

This here-and-now quality distinguishes traumatic memories from intrusive memories that occur in many psychological disorders, including depression and generalized anxiety disorder (GAD) (Brewin et al., 2010). Traumatic memories are typically experienced as vivid, spontaneous, and uncontrollable, and sometimes involve dissociation (Brewin, 2025). Trauma-focused therapies directly target these memory disturbances to reduce PTSD symptoms by helping patients confront the feared and avoided trauma memories, reprocess the sensory and emotional experiences, and integrate contextual information to form a coherent narrative of the event. This helps embed the episodic memory into autobiographical memory, thereby alleviating re-experiencing symptoms and, consequently, reducing the sense of current threat and avoidance of trauma reminders. Trauma-focused therapies are highly effective (e.g., Asmundson et al., 2019; Cusack et al., 2016; Lenz et al., 2017; McLean et al., 2022; Watts et al., 2013), with meta-analytic evidence showing ‘large’ reductions in PTSD symptoms relative to controls (*g* = 1.27) (Cuijpers et al., 2025).

PTSD typically co-occurs with other psychological disorders, including depression and GAD, even when adjusting for overlapping symptoms (Elhai et al., 2011; Elhai et al., 2008; Kessler et al., 1995). Furthermore, trauma-focused therapies for PTSD produce large reductions in co-occurring depression and anxiety symptoms (e.g., Powers et al., 2010; Thielemann et al., 2022), including interventions that focus exclusively on reprocessing traumatic memories (e.g., Sloan et al., 2025; Thompson-Hollands et al., 2018). Although the mechanisms underlying these improvements in comorbid depression and anxiety symptoms are not fully understood, several explanations have been proposed (see Horesh et al. 2017 for a review). These are illustrated in Fig. 1 (n.b., these explanations have focused on the relations between PTSD and depression but can be extended to GAD).

**Fig. 1:**
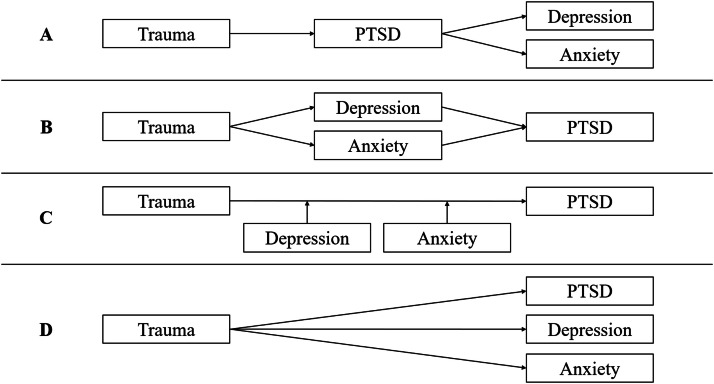
Four possible pathways linking trauma exposure (and the development of traumatic memories) to posttraumatic stress disorder (PTSD), depression, and anxiety. Panel A depicts a model in which trauma leads to PTSD, which in turn increases the likelihood of developing depression and/or anxiety. Panel B shows trauma initially leading to depression and/or anxiety, which thereafter increases the risk of developing PTSD. Panel C illustrates a model in which pre-existing depression and/or anxiety prior to trauma exposure increases the risk of developing PTSD. Panel D depicts a model in which trauma exposure contributes independently to PTSD, depression, and anxiety via a shared risk factor or set of risk factors. Adapted from Horesh et al. (2017).

One explanation is that trauma leads to PTSD which then increases the likelihood of developing depression and/or GAD (panel A). A second is that trauma causes depression or GAD which then increases the likelihood of developing PTSD (panel B). A third is that pre-existing depression and/or GAD increases the risk of developing PTSD following trauma (panel C). Finally, a fourth explanation is that trauma causes PTSD, depression, and/or GAD to develop simultaneously because of a shared risk factor or set of risk factors (panel D). Empirical evidence testing these competing explanations is sparse, and findings are mixed (Dekel et al., 2014; Horesh et al., 2015). While all four explanations likely have some basis in reality, the focus of this study is on the final explanation.

Research indicates that PTSD, depression, and GAD symptoms develop in close temporal proximity following trauma, suggesting a potential shared risk-factor (e.g., Amouzadeh Ghadikolai et al., 2025; Breslau, 2002; Bryant et al., 2010; Horesh et al., 2017; McFarlane & Papay, 1992; O’Donnell et al., 2004; Shalev et al., 1998). Few candidates have been suggested, but one possibility is that the memory disturbances underlying PTSD might also contribute to the development of depression and GAD (Abramovitch et al., 2021; East-Richard et al., 2020). Several lines of evidence support this possibility.

First, memory disruptions observed in depression closely resemble those in PTSD (Dillon & Pizzagalli, 2018; LeMoult & Gotlib, 2019). Patients with depression often have a history of chronic stress preceding the onset of a depressive episode, and chronic stress affects hippocampal functioning while concurrently sensitizing the amygdala, resulting in impaired memory consolidation and an overgeneralized fear response. Dysregulation of episodic memory has also been documented in various anxiety disorders (Airaksinen et al., 2005; Moran, 2016). Second, although many individuals with depression and/or GAD have no history of trauma, trauma exposure is a robust risk-factor for both disorders (Hogg et al., 2023; Li et al., 2016; Liu et al., 2025; Mandelli et al., 2015; McKay et al., 2021), particularly recent or early-life trauma, both of which are associated with treatment-resistant depression (Fantasia et al., 2025; Tunnard et al., 2014). Consistent with this, trauma-focused therapies have been shown to effectively treat depression and anxiety even in the absence of PTSD (Carletto et al., 2021; Fracalanza et al., 2014; Kroener et al., 2023; Ma & Lo, 2022; Sepehry et al., 2021; Yunitri et al., 2020), with one meta-analysis finding that trauma-focused cognitive-behaviour therapies outperformed non-trauma-focused cognitive-behaviour therapies for depression (Dominguez et al., 2021). Together, these findings suggest that trauma-related memory disturbances, known to contribute to the development of PTSD symptoms, may also play a role in the development of depression and/or GAD symptoms.

Ideally, prospective longitudinal data collected before and after a traumatic event would be gathered to ascertain *when* PTSD, depression, and GAD symptoms emerge, *how* they influence one another, and *if* traumatic memories predict their onset. In the absence of such data, it is still possible to test predictions about the relations between these variables using cross-sectional data. For example, if traumatic memories cause PTSD symptoms which then cause depression and/or GAD symptoms (Panel A, Fig. 1), there should be no direct effect of traumatic memories on depression and/or GAD symptoms, and any observed association would reflect the unmodelled effects of PTSD symptoms. Thus, with cross-sectional data, traumatic memories should be positively and significantly correlated with depression and GAD symptoms in a bivariate analysis, but these associations should become non-significant when posttraumatic stress symptoms are modelled (i.e., PTSD and DSO symptoms in the *ICD-11* framework). However, if traumatic memories independently contribute to depression and/or GAD symptoms (Panel D, Fig. 1), a direct effect should be observed. With cross-sectional data, positive associations between traumatic memories and depression and GAD symptoms should remain statistically significant after adjusting for posttraumatic stress symptoms. This study tested these competing hypotheses.

## Methods

### Participants

This study uses data from two general adult population samples, one from Ukraine (*N* = 2050) and one from the United Kingdom of Great Britain and Northern Ireland (UK: *N* = 975). Both samples were collected by survey research companies with access to multiple national panels for online research. The Ukrainian data were collected by TGM Research between September 7 and 18, 2023, and the UK data were collected by Qualtrics between March 1 and 27, 2024. In both cases, quota sampling methods were used to construct a sample that represented the general adult population of each nation across several sociodemographic variables. In Ukraine, the quota sampling variables were sex, age, and current living location (north, south, east, west, and central Ukraine), while in the UK they were sex, age, income level, and UK nation (England, Scotland, Wales, and Northern Ireland). Since these samples were constructed using non-probability-based methods, they are not fully representative of the populations from which they were drawn.

To ensure data quality, both surveys included attention checks, and screening procedures were employed to identify and exclude invalid responses such as those with unusually fast completion times, suspicious response patterns, and duplicate IP addresses. Ethical approval for the collection of the Ukrainian data was granted by the SI Institute of Psychiatry, Forensic Psychiatric Examination and Drug Monitoring of the Ministry of Health of Ukraine, and for the UK data from the Social Research Ethics Committee at Maynooth University, Ireland. Demographic information for both samples is presented in Table 1.

**Table 1:** Sociodemographic details for both (total) samples.

Ukraine (*N* = 2050)	%	UK (*N* = 975)	%
**Sex**		**Sex**	
Female	48.3%	Female	51.5%
Male	51.7%	Male	48.5%
**Age**		**Age**	
18-29	20.8%	18-29	20.2%
30-39	25.2%	30-39	21.9%
40-49	23.2%	40-49	16.1%
50-59	19.0%	50-59	15.5%
60+	11.8%	60+	26.3%
**Birthplace**		**Birthplace**	
Ukraine	92.0%	UK	89.3%
**Region of Ukraine**		**Region of UK**	
Western Ukraine	24.3%	England	86.4%
North Ukraine	22.0%	Wales	4.9%
Central Ukraine	13.5%	Scotland	6.8%
Eastern Ukraine	15.6%	Northern Ireland	1.9%
South Ukraine	24.7%		
**Living Location**		**Living Location**	
Rural Area	18.9%	Rural Area	34.5%
Urban Area	81.1%	Urban Area	65.5%
**Highest Education**		**Highest Education**	
Completed Mandatory Schooling	2.8%	No Qualification	4.0%
Completed Secondary School	10.9%	O-level / GCSE or similar	25.3%
Completed Vocational School	28.4%	A-level or similar	28.5%
Completed University	57.9%	Undergraduate Degree	28.9%
		Postgraduate Degree	13.2%
**Employment Status**		**Employment Status**	
Full-time (self)/employed	47.3%	Full-time (self)/employed	45.8%
Part-time (self)/employed	19.6%	Part-time (self)/employed	17.4%
Retired	10.3%	Retired	18.8%
Unemployed	9.1%	Unemployed (Seeking Work)	5.2%
Temporarily Unemployed (Due to War)	7.8%	Unemployed (Not Seeking Work)	4.6%
Student	3.5%	Student	3.1%
Disability (Temporary or Permanent)	2.5%	Disability (Temporary or Permanent)	5.0%
**Relationship Status**		**Relationship Status**	
In A Committed Relationship	64.9%	In a committed relationship	71.2%
Not in a committed relationship	35.1%	Not in a committed relationship	28.8%
**Do You Have Children**		**Do You Have Children**	
Yes	69.5%	Yes	64.6%

### Measures

**Trauma exposure**: All participants completed the *International Trauma Exposure Measure* (Hyland et al., 2021) which assesses lifetime exposure to 21 events consistent with the *ICD-11* definition of a traumatic event. Participants indicated whether they had experienced each event by responding either *‘Yes’* (1) or *‘No’* (0). Total scores range from 0 to 21, with higher scores reflecting exposure to a greater number of different traumatic events. Participants also identified the event that currently troubles them the most (i.e., their index trauma) and how long ago it occurred. The predictive validity of the scale scores has been demonstrated in different samples (Hyland et al., 2021; Rossi et al., 2022).

***ICD-11* PTSD and CPTSD**: Participants who reported experiencing one or more traumatic events completed the *International Trauma Questionnaire* (ITQ: Cloitre et al., 2018). The ITQ is the most widely used self-report measure of *ICD-11* PTSD and CPTSD symptoms. Participants were instructed to answer all scale items thinking about their previously identified index trauma. The ITQ has 12 items: six measuring the three PTSD symptom clusters by two items each, and six items measuring the three DSO symptom by two items each. Participants indicated how bothered they have been by these symptoms on a five-point Likert scale ranging from *‘Not at all’* (0) to *‘Extremely’* (4). Total scores range from 0–24 for both the PTSD and DSO symptoms, with higher scores reflecting greater symptom severity. Much evidence supports the psychometric properties of the ITQ scores (Redican et al., 2021), including the Ukrainian translation (Ho et al., 2023). In this study, the internal reliability estimates were good in the Ukrainian (PTSD items, *α* = 0.84, DSO items, *α* = 0.88) and the UK (PTSD items, *α* = 0.88, DSO items, *α* = 0.90) samples.

**Traumatic memories**: Participants who experienced a traumatic event and reported a non-zero score on one of the two ITQ items measuring re-experiencing symptoms (flashbacks or nightmares) completed the *Experiences of Traumatic Memories Questionnaire* (ETMQ: Hyland et al., 2024a). The ETMQ is an eight-item self-report measure assessing some of the most common and defining phenomenological experiences of traumatic memories such as uncontrollability, vividness, and accompanying dissociation and somatic reactions (Hyland et al., 2023; Hyland et al., 2025). Participants were given the following instruction: *In the previous section you indicated having powerful images or memories that sometimes come into your mind in which you feel the experience is happening again inthehereandnow? Please take a moment to think of the most prominent image or memory that you re-experience as happening again in the present*’. Participants then indicated how true each item was for them using a five-point Likert scale ranging from ‘*Almost never true*’ (0) to ‘*Almost always true*’ (4). The possible range of scores is 0–32 with higher scores indicating more intense experiences of traumatic memories. The internal reliability of the scale scores was good in the Ukrainian (*α* = 0.88) and UK (*α* = 0.88) samples.

***ICD-11* depression and GAD:** All participants completed the *International Depression Questionnaire* (IDQ: Shevlin et al., 2023) and the *International Anxiety Questionnaire* (IAQ: Shevlin et al., 2023) which are self-report measures of *ICD-11* depressive episode and GAD. Participants indicated how frequently they experienced the nine symptoms of depression over the last two weeks, and the eight symptoms of GAD over the last several months, using a five-point Likert scale ranging from *‘Never’* (0) to *‘Every Day’* (4). Total IDQ scores range from 0–36, and total IAQ scores from 0–32, with higher scores on each scale reflecting greater symptom severity. The psychometric properties of both scales are well supported (e.g., Alpay et al., 2023; Hyland et al., 2024b), and the internal reliability of the scale scores in the Ukrainian (IDQ, *α* = 0.93; IAQ, *α* = 0.93) and UK (IDQ, *α* = 0.94; IAQ, *α* = 0.93) samples was excellent.

### Data analysis

Descriptive statistics were used to summarise scores for traumatic memories and symptoms of PTSD, DSO, depression, and GAD in each sample. The Pearson product-moment correlation test was used to determine the bivariate associations between these variables. Hierarchical multiple regression analysis was used to test whether traumatic memories explained unique variance in depression and GAD symptoms beyond posttraumatic stress symptoms, with variables entered in a theoretically informed manner. Two analyses were performed with depression and GAD symptom scores as criterion variables. In the first step of the model, traumatic memories were entered to calculate their unadjusted association with depression and GAD symptoms, respectively. In step 2 of the model, a total PTSD symptom variable was added to determine if traumatic memories remained associated with each criterion variable adjusting for the ‘core’ posttraumatic stress symptoms. In step 3, a total DSO symptom variable was added to the model to determine if traumatic memories remained associated with each criterion variable adjusting for the core posttraumatic stress symptoms and the broader DSO symptoms. As a sensitivity check, all analyses were run with time since index trauma added as a covariate at Step 1 to examine whether recency of trauma exposure influenced the pattern of associations. The analyses were performed independently in the Ukrainian and UK datasets, and all analyses were conducted using SPSS version 29.

## Results

In the Ukrainian sample, 90.0% (*n* = 1845) were exposed to at least one trauma, and 70.7% of the full sample (*n* = 1450) had a non-zero score on at least one of the two re-experiencing symptoms and were therefore invited to complete the traumatic memories questionnaire. In the UK sample, 72.5% (*n* = 707) were exposed to at least one trauma, and 53.6% of the full sample (*n* = 523) met the same re-experiencing symptom criterion. All subsequent analyses were based on these participants. Descriptive statistics for the main study variables are presented in Table 2. All variables were positively skewed in each sample, which is understandable as these are samples drawn from the general population.

**Table 2:** Descriptive statistics for all study variables.

	Mean (95% CIs)	Median	SD	Range
**Ukraine (*n* = 1450)**				
Trauma Memories	10.89 (10.54-11.24)	10	6.83	0-32
PTSD	8.37 (8.13-8.60)	8	5.14	0-24
DSO	6.70 (6.46-6.95)	5	5.31	0-24
Depression	8.45 (8.13-8.78)	7	7.56	0-36
GAD	10.31 (9.98-10.63)	9	7.47	0-32
**UK (*n* = 523)**				
Trauma Memories	14.77 (14.15-15.39)	15	7.22	0-32
PTSD	7.74 (7.30-8.18)	7	5.95	0-24
DSO	8.06 (7.60-8.52)	7	6.23	0-24
Depression	9.22 (8.68-9.77)	7	8.70	0-36
GAD	10.63 (10.13-11.12)	9	7.82	0-32

*Note.* CIs = Confidence Intervals; SD = Standard Deviation; PTSD = Posttraumatic Stress Disorder; DSO = Disturbances in Self Organization; GAD = Generalized Anxiety Disorder

The bivariate correlations are presented in Table 3. All variables were significantly (*p* < 0.001), positively, and strongly correlated with one another. In both samples, traumatic memories correlated with PTSD, DSO, depression, and GAD symptoms at similar magnitudes.

**Table 3:** Pearson correlations between all variables.

	1	2	3	4	5
**Ukraine (*n* = 1450)**					
1. Trauma Memories	1				
2. Posttraumatic stress disorder	.57⁎⁎⁎	1			
3. Disturbances in self-organization	.64⁎⁎⁎	.59⁎⁎⁎	1		
4. Depression	.59⁎⁎⁎	.56⁎⁎⁎	.67⁎⁎⁎	1	
5. Generalized anxiety disorder	.59⁎⁎⁎	.60⁎⁎⁎	.64⁎⁎⁎	.86⁎⁎⁎	1
**UK (*n* = 523)**					
1. Trauma Memories	1				
2. Posttraumatic stress disorder	.59⁎⁎⁎	1			
3. Disturbances in self-organization	.62⁎⁎⁎	.63⁎⁎⁎	1		
4. Depression	.58⁎⁎⁎	.57⁎⁎⁎	.79⁎⁎⁎	1	
5. Generalized anxiety disorder	.59⁎⁎⁎	.61⁎⁎⁎	.78⁎⁎⁎	.88⁎⁎⁎	1

⁎⁎⁎ *p* < 0.001.

The results of the hierarchical multiple regression analyses are presented in Tables 4 (Ukraine) and 5 (UK). In the Ukrainian sample, at step 1, traumatic memories was significantly associated with depression (*β* = 0.59, *p* < 0.001) and GAD (*β* = 0.59, *p* < 0.001) symptoms, accounting for 34.9% of the variance in depression symptoms (*F*(1, 1448) = 776.13, *p* < 0.001) and 34.3% in GAD symptoms (*F*(1, 1448) = 754.88, *p* < 0.001).

**Table 4:** Hierarchical multiple regression model predicting symptoms of depression and generalized anxiety disorder (GAD) in the Ukrainian sample (*n* = 1450).

	Depression symptoms			GAD symptoms		
	R^2^	ΔR^2^	β	R^2^	ΔR^2^	β
**Step 1**	.35***	–		.34***	–	
Trauma Memories			.59***			.59***
**Step 2**	.42***	.07***		.44***	.10***	
Trauma Memories			.41***			.36***
PTSD			.32***			.39***
**Step 3**	.52***	.10***		.50***	.06***	
Trauma Memories			.21***			.21***
PTSD			.18***			.27***
DSO			.43***			.34***

*Note.* R^2^ = percentage of variance explained; ΔR^2^ = change in the percentage of variance explained; β = standardized beta value; PTSD = posttraumatic stress disorder; DSO = disturbances in self-organization; Statistical significance: *p < 0.05; **p < 0.01; ***p < 0.001.

**Table 5:** Hierarchical multiple regression model predicting symptoms of depression and generalized anxiety disorder (GAD) in the UK sample (*n* = 523).

	Depression symptoms			GAD symptoms		
	R^2^	ΔR^2^	β	R^2^	ΔR^2^	β
**Step 1**	.34***	–		.34***	–	
Trauma Memories			.58***			.59***
**Step 2**	.42***	.08***		.43***	.10***	
Trauma Memories			.37***			.35***
PTSD			.35***			.40***
**Step 3**	.65***	.23***		.62***	.19***	
Trauma Memories			.11**			.11**
PTSD			.08*			.16***
DSO			.67***			.61***

*Note.* R^2^ = percentage of variance explained; ΔR^2^ = change in the percentage of variance explained; *β* = standardized beta value; PTSD = Posttraumatic Stress Disorder; DSO = Disturbances in Self-Organization; Statistical significance: *p < 0.05; **p < 0.01; ***p < 0.001.

The inclusion of PTSD symptoms at step 2 explained an additional 7.1% of variance in depression symptoms (ΔR² = 0.071; ΔF(1, 1447) = 177.18, *p* < 0.001) and 10.2% of variance in GAD symptoms (ΔR² = 0.102; ΔF(1, 1447) = 266.48, *p* < 0.001). The associations between traumatic memories and depression (*β* = 0.41, *p* < 0.001) and GAD (*β* = 0.36, *p* < 0.001) symptoms were attenuated but remained robust and statistically significant.

Adding DSO symptoms at step 3 explained a further 9.6% of variance in depression (ΔR² = 0.096; ΔF(1, 1446) = 286.41, *p* < 0.001) and 6.1% of variance in GAD (ΔR² = 0.061; ΔF(1, 1446) = 177.44, *p* < 0.001) symptoms. Traumatic memories remained significantly associated with depression (*β* = 0.21, *p* < 0.001) and GAD (*β* = 0.21, *p* < 0.001) symptoms when adjusting for PTSD and DSO symptoms.

In the UK sample, at step 1, traumatic memories was significantly associated with depression (*β* = 0.58, *p* < 0.001) and GAD (*β* = 0.59, *p* < 0.001) symptoms, explaining 33.5% of the variance in depression symptoms (*F*(1, 521) = 262.35, *p* < 0.001) and 34.3% of variance in GAD symptoms (*F*(1, 521) = 272.15, *p* < 0.001).

When PTSD symptoms were added at step 2, a further 7.7% of variance in depression (ΔR² = 0.077; ΔF(1, 520) = 68.48, *p* < 0.001) and 10.3% of variance in GAD (ΔR² = 0.103; ΔF(1, 520) = 96.62, *p* < 0.001) was explained. Traumatic memories remained significantly associated with depression (*β* = 0.37, *p* < 0.001) and GAD (*β* = 0.35, *p* < 0.001) symptoms.

The addition of DSO symptoms at step 3 explained a further 23.1% of variance in depression (ΔR² = 0.231; ΔF(1, 519) = 335.22, *p* < 0.001) and 19.1% of variance in GAD (ΔR² = 0.191; ΔF(1, 519) = 272.49, *p* < 0.001) symptoms. Traumatic memories remained significantly associated with depression (*β* = 0.11, *p* < 0.001) and GAD (*β* = 0.11, *p* < 0.001) symptoms.

When time since index trauma was included as a covariate, beta coefficients remained unchanged in most cases and only negligible fluctuations (±.01) were observed in a small number of cases, indicating that these findings were not influenced by trauma recency.

## Discussion

The primary aim of this study was to test if traumatic memories were associated with symptoms of depression and GAD independently of posttraumatic stress symptoms. Current results, derived from two culturally distinct samples, indicated that traumatic memories were not only strongly associated with depression and GAD symptoms in a bivariate analysis, but that these positive associations remained when adjusting for the effects of PTSD and DSO symptoms. These findings are consistent with the proposition that traumatic memories are directly associated with depression and GAD symptoms, independent of the influence of posttraumatic stress symptoms, and may therefore represent a shared risk factor for these disorders.

Theoretically, these findings suggest that depression and GAD may be, partly, trauma-related disorders. Current psychiatric nomenclature draws a clear distinction between ‘stress and trauma-related disorders’ like PTSD, and other disorders like depression and GAD which do not require trauma exposure for diagnosis. While depression and GAD can develop without a history of trauma, substantial evidence indicates that these disorders – and many others – are more likely to develop when there is a history of trauma (Hogg et al., 2023; Li et al., 2016; Liu et al., 2025; Mandelli et al., 2015; McKay et al., 2021). Current results extend upon these findings by elucidating one psychological mechanism through which trauma may contribute to the development of depression and GAD (i.e., disturbances in memory). More broadly, these results suggest that there may not be as clear a demarcation between trauma- and non-trauma-related disorders as current psychiatric nomenclature implies. While these categorisations are clearly made for pragmatic reasons, it may have the unintended consequence of obscuring an understanding of the key role that trauma can play in the development, at least some of the time, in disorders not explicitly described as trauma related.

Current results, if borne out by future research, would make sense of several clinical observations. Trauma-focused therapies have been shown to not only reduce symptoms of depression and anxiety that co-occur with PTSD (Powers et al., 2010; Sloan et al., 2025; Thielemann et al., 2022; Thompson-Hollands et al., 2018), but to effectively treat depression and anxiety when there is no concurrent PTSD diagnosis (Carletto et al., 2021; Dominguez et al., 2021; Fracalanza et al., 2014; Kroener et al., 2023; Ma & Lo, 2022; Sepehry et al., 2021; Yunitri et al., 2020). If traumatic memories have no direct influence on depression and GAD symptoms, these observations are difficult to explain. Contrastingly, if traumatic memories have a direct influence on symptoms of PTSD, depression, and GAD, all of these clinical observations are easily understandable; resolution of a shared risk factor brings about simultaneous symptom relief across all three disorders. It is a parsimonious explanation of the observed data.

Another clinical implication relates to the importance of screening for trauma exposure and memory disturbances in individuals presenting with symptoms of depression and GAD, even in the absence of problems that might suggest the relevance of a PTSD diagnosis. Without routine screening for a history of trauma, one important psychological mechanism might go unrecognised, and therefore untreated. Relatedly, reprocessing of traumatic memories is not currently a component of widely used, evidenced-based psychotherapies for depression and GAD, but current findings suggest that such an approach may be warranted for individuals with a history of trauma and related memory difficulties.

Finally, the current results raise the possibility that some cases of treatment resistant depression (and GAD) might be effectively treated by reprocessing traumatic memories. About 30% of people with depression do not respond to standard evidence-based treatments (McIntyre et al., 2023), and one of the most consistent factors associated with treatment resistant depression is a history of trauma, particularly early life trauma (Fantasia et al., 2025; Tunnard et al., 2014). If traumatic memories are playing a key role in the development and maintenance of depression for some people, then treatment would not be expected to be successful unless these memory problems are resolved.

Given the theoretical and clinical implications of these findings, it is important to emphasise the cross-sectional nature of the data used. This study has not demonstrated that traumatic memories have a causal effect on depression or GAD symptoms, nor that traumatic memories predict the future onset of depression and GAD symptoms. Rather, it has simply found that the pattern of associations between traumatic memories and depression, GAD, and PTSD/DSO symptoms are consistent with the proposition that traumatic memories are directly associated with depression and GAD symptoms, independent of PTSD/DSO symptoms, and therefore may represent a shared risk factor for these disorders.

In addition to the cross-sectional nature of the data, there are some other limitations that should be noted. First, the use of non-probability sampling limits the generalizability of the findings to the broader populations of Ukraine and the UK (and other countries). Second, the two samples were drawn from markedly different contexts; Ukraine was in a state of war when the data were collected, whereas the UK was not. While this can complicate interpretations of findings across the samples, this can also be considered a strength of the study given that similar findings were observed in very different contexts. Third, posttraumatic stress symptoms were measured in accordance with the *ICD-11* description, which is quite different to the *DSM-5-TR* description of PTSD that includes symptoms such as loss of pleasure/interest, irritability, and concentration and sleeping difficulties that overlap considerably with depression and GAD (American Psychiatric Association, 2022). Whether current results would replicate with the *DSM-5-TR* model of PTSD is both unknown, and a complicated matter given the degree of symptom overlap.

In conclusion, this study provides evidence that traumatic memory disturbances are associated with depression and GAD symptoms, independent of the influence of posttraumatic stress symptoms. These findings are consistent with the view that posttraumatic stress, depression, and GAD symptoms share a common risk factor, and that dysfunction in episodic and autobiographical memory is one such shared risk factor. If disturbance in memory caused by a traumatic event is indeed a shared risk factor, it opens up the possibility of using existing, trauma-focused psychotherapeutic techniques to improve treatment outcomes for depression and GAD.

## Author statement and acknowledgements

All authors have made substantial contributions to this study, including its conception and design, data acquisition, analysis and interpretation of data, drafting and revising the article, and approving the final version submitted. The first author, Marcus Broughill, was supported by a National University of Ireland (NUI) Travelling Doctoral Studentship. The authors have no other acknowledgements to declare.

## Funding statement

Marcus Broughill was supported by a National University of Ireland (NUI) Travelling Doctoral Studentship.

## Declaration of competing interest

The authors declare that they have no known competing financial interests or personal relationships that could have appeared to influence the work reported in this paper.
